# Initis or Nutrition and Exercises
**Initis or Nutrition and Exercises.* By A. Rabagliati, M.A., M.D. London: C. W. Daniel, Ltd. 1916. Pp. 183 + xi. Price 10s. 6d. net.


**Published:** 1917-08-11

**Authors:** 


### INITIS OR NUTRITION AND EXERCISES.*

Dr. A. Rabagliati, of Bradford, has conceived the notion that a number of conditions of ill-health are due to congestion of the "strong or connective tissues of the body"; and he proposes to group these conditions under the term "initis." According to its sponsor, "initis" has a highly comprehensive range. It includes, together with others, neurasthenia or "weakness of the nerves," hysteria or "wombiness," general debility, rheumatic neuralgia, neuralgic rheumatism, peliosis, and "uric-acid-æmia or oxy-urichæmia." At one extreme, congestion of the connective tissues explains "why young women, and sometimes older ones, burst out a-crying"; at the other, it is the highway to tuberculosis, Bright's disease, apoplexy, cancer, angina pectoris, and insanity. Happily, there is, however, a relatively simple plan of salvation, for the prevention of "initis" and of its evil sequences may be found in the revision of our food habits, and in the practice of systematic exercises. Such, so far as we can follow Dr. Rabagliati, are the propositions which he professes to teach by the publication of his book. Unfortunately, instead of being content to submit a plain statement of his case and of the evidence on which he relies, he has swamped his story with a confused mass of meaningless verbiage, inaccurate physiology, and more than doubtful philosophy. His chapters are crowded with the superficial analogies and ponderous phrases which are the mark of the pseudo-scientific habit of mind, and his book must be awarded high rank in this order of writing. To take but two examples, Dr. Rabagliati wishes to assure his readers that were his advice followed the term of human life would be extended, and to announce this prophecy he writes: "The human body would remain the normal habitation of anthropino-zoo-dynamic for a very much longer time than it does now." And again, "just as the force of gravitation or hylo-dynamic seems to procreate the ether as the vehicle or means by which it may convey itself throughout the infinite space subject to its authority, so zoo-dynamic or the force of animal life seems to introduce or procreate the connective tissue as the vehicle or means by which the whole body (and every organised body) shall be subject to the authority of the life force." If this sort of wordy twaddle is intended for the medical profession, Dr. Rabagliati, we fancy, has mistaken his market. Possibly there may exist in some suburban or sylvan retreat an audience which in all innocence will regard these pages as the product of deep learning and profound thought. But such credulous souls will hardly be found in professional circles. As for the hardened reviewer, he knows well exhibitions of this order. Hence, after a moderate number of pages, he seeks—tobacco failing—refuge in sleep, and may if he is fortunate find on awaking that the book, appropriately enough, has fallen into the waste-paper basket. In at least one such instance no attempt at rescue will be reported.

We must say a word about the illustrations. These are numerous. They represent ladies and gentlemen in various postures pointing, without any restraint from an unscientific convention, to certain portions of their bodies and credited with exercising pressure thereon. Two of the most impressive are photographs of men who by applying the tips of the fingers to the front of the breastbone are, we are told, successfully defying attacks of angina pectoris. In one instance the effort is a two-handed one; while in the other success is achieved by the right hand alone; and it is to this latter performer that we personally should adjudge the prize. Pictorial representations of modern success are made all the brighter by sad stories of ancient failure. Marcus Aurelius, we read, died at fifty-nine years of age "because, Galen notwithstanding, he did not understand nutrition." Fronto, "the Emperor's beloved tutor," seems to have been in no better case, and accordingly he suffered from gout. Even Galen himself only reached seventy years of age, and a rumour that he attained one hundred and forty years is to be discredited; as he did not "understand nutrition," the suggestion is obviously absurd. Poor Pliny the Elder, again, as a consequence of similar ignorance, became so wheezy and stout that the volcanic eruption at Herculaneum proved too swift for him. How different would have been these histories had "initis" dawned at an earlier day, and emperors and tutors and physicians been protected from congestion of their connective tissues! Now they have become the mere stuff by which the thin pages of a modern writer may be padded out into a pretentious volume. It is a hard fate, and we trust that justice will protect them from the aggravation of a second edition.

* *Initis or Nutrition and Exercises.* By A. Rabagliati, M.A., M.D. London: C. W. Daniel, Ltd. 1916. Pp. 183 + xi. Price 10s. 6d. net.

